# Supplementary material for: TDP-43 interacts with amyloid-β, inhibits fibrillization, and worsens pathology in a model of Alzheimer’s disease
Source: Nat Commun. 2020 Nov 23;11:5950. doi: 10.1038/s41467-020-19786-7 (PMC7683652; doi:10.1038/s41467-020-19786-7)
Supplement: Supplementary file 1 — Supplementary Information [file 41467_2020_19786_MOESM1_ESM.pdf]

Supplementary Information

TDP-43 interacts with amyloid-β, inhibits fibrillization, and worsens pathology in a model of Alzheimer's disease

Yao-Hsiang Shih[1,2,3§], Ling-Hsien Tu[1,4§], Ting-Yu Chang[1,5], Kiruthika Ganesan[1], Wei-Wei Chang[1], Pao- Sheng Chang[1], Yu-Sheng Fang[1,6], Yeh-Tung Lin[1,5], Lee-Way Jin[7], and Yun-Ru Chen[1,5*]

Supplementary Method

Size-exclusion chromatography. TSKgel G4000SWXL column was first standardized by calibration kits (GE Healthcare Life Science) using molecular weight standards including thyroglobulin (669 kDa), ferritin (440 kDa), aldolase (158 kDa), conalbumin (75 kDa), ovalbumin (44 kDa), and ribonuclease A (13.7 kDa). A volume of 20 μL of fresh dialyzed and filtered TDP-43 proteins was injected into column and elution time was recorded. The running buffer contains 0.3 M NaCl in 0.1 M sodium phosphate buffer, pH 7.0, and the flow rate was 0.5 mL/min. Data were plotted in GraphPad Prism 7.0.

Western blot for Aβ and TDP-43 in AD brains. Western blot was performed to observe Aβ assembly in AD brain tissues. The Aβ assembly in the extracellular-enriched fractions of hippocampi were examined following aforementioned protocol. Each well was loaded with 35 μg of total protein. tissues, The Triton-insoluble parts were further extracted by urea buffer (7 mol/L urea, 2 mol/L thiourea, 4% 3-[(3-cholamidopro-pyl)dimethylammonio]-1-propanesulfonate (CHAPS), 30 mmol/L Tris-HCl, pH 8.5)[1] and the hyperphosphorylated TDP-43 in urea fractions of hippocampi were detected by anti-TDP-43 phospho Ser409/410 antibody (1:1000, Cosmo Bio, CA, USA) and goat anti-mouse IgG-HRP (1:5000, Genetex).

Supplementary Table

Supplementary Table 1. Primers used for cloning.

Clone Name	Forward Primer	Reverse Primer
TDP-43_265	5'TAGGATCCGGCTGCTAACAAAGCCC3'	5'ATTGTGCTTAGGTTCGGCATTGGATA3'

Supplementary Figures

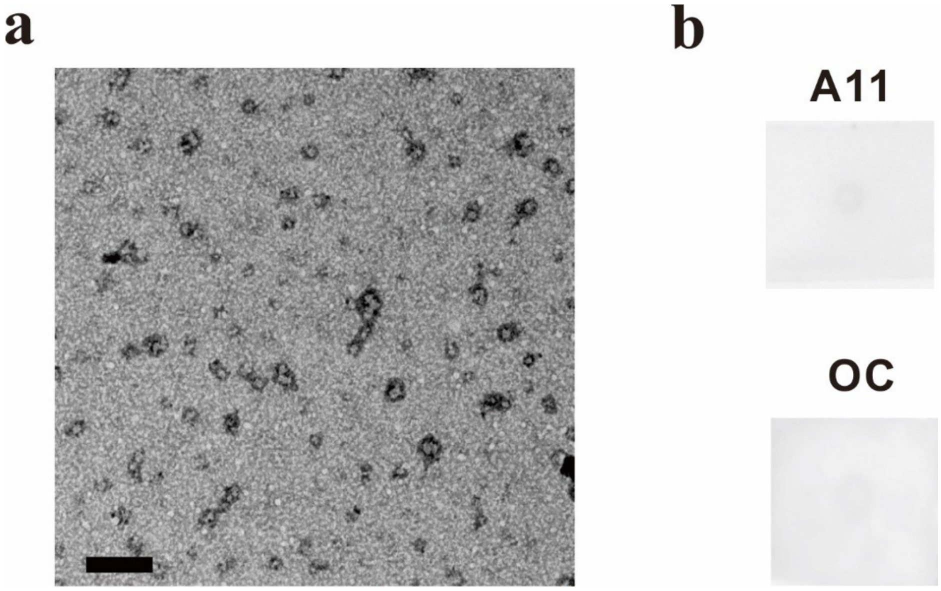

Supplementary Figure 1. **TEM image and epitope of TDP-43 oligomers.** (a) TEM image of recombinant TDP-43 oligomers. Scale bar represents 100 nm. More than five times of independent experiments were performed. (b) Dot blot of TDP-43 oligomers probed by A11 and OC conformational antibodies. Three times of independent experiments were performed. Source data are provided as a Source Data file.

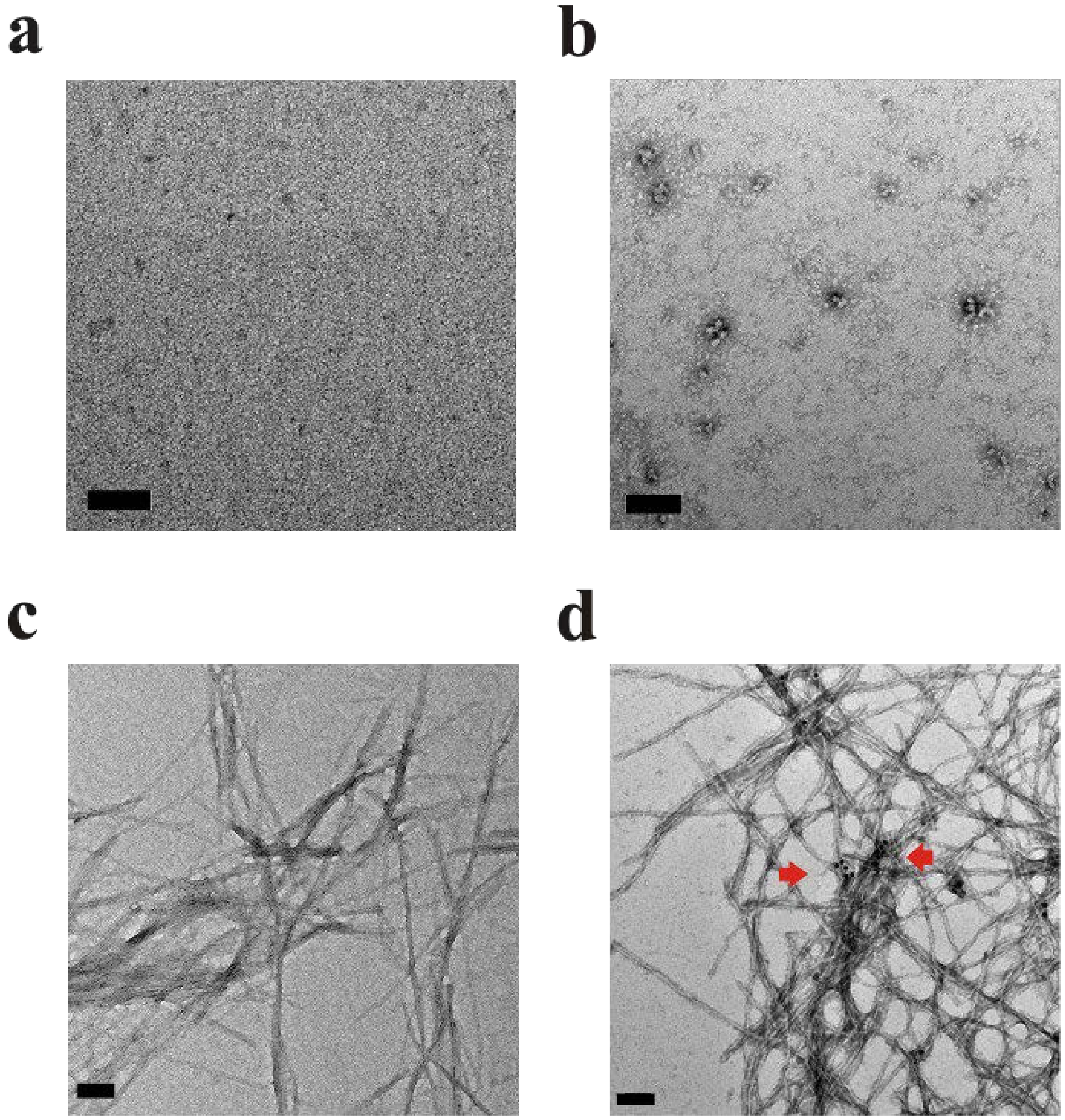

Supplementary Figure 2. **TEM images of different Aβ status**. TEM images of (a) Aβ monomers, (b) Aβ oligomers, and (c) Aβ fibrils. (d) Immunogold labeling of TDP-43 with Aβ fibrils. TDP-43 probed by anti-TDP-43 N-term antibody is indicated by red arrow. The scale bars are 100 nm. Two times of independent experiments were performed. Source data are provided as a Source Data file.

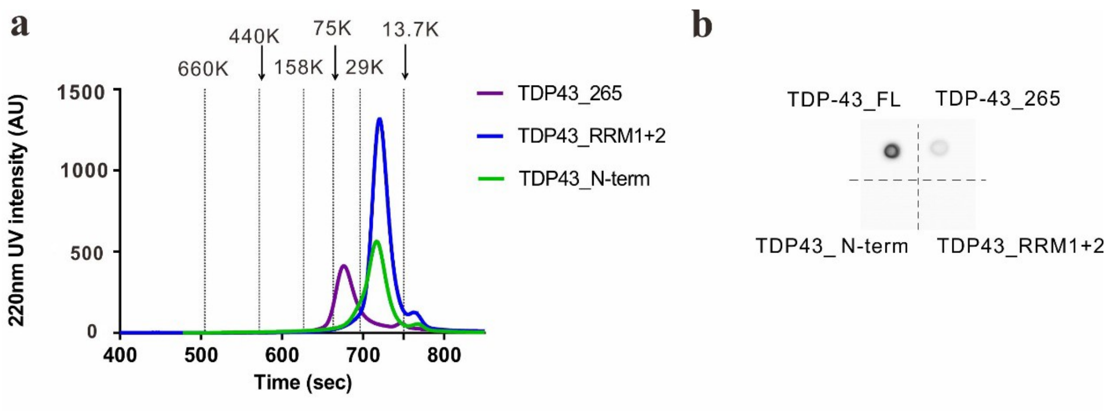

Supplementary Figure 3. **Assembly and properties of TDP-43 variants.** (a) Size-exclusion chromatography of TDP-43 variants. The results suggest that TDP-43_265 (purple) and TDP-43_N-term (green) are homodimers and TDP-43_RRM1+2 (blue) is a monomer. (b) Dot blots of TDP-43 variants by TDP-43 oligomer specific antibody. Freshly dialyzed full- length TDP-43 and its truncated forms were dotted on nitrocellulose membranes and subjected to dot blotting. The membrane was probed by polyclonal antibody TDP-O that specifically recognized TDP-43 oligomers [2]. Source data are provided as a Source Data file.

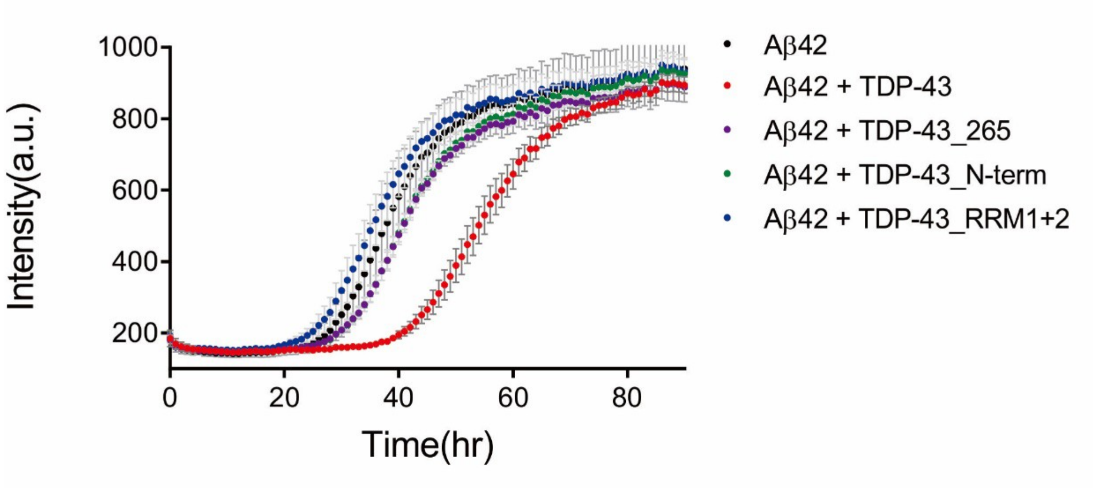

Supplementary Figure 4. Full-length TDP-43 inhibited Aβ42 fibrillization. (a) ThT assay of Aβ42 fibrillization (control, black) and with full-length TDP-43 (red), TDP-43_265 (purple), TDP-43_N-term (green), TDP-43_RRM1+2 (blue) in 10 mM Tris buffer, pH 8.0. Aβ42 concentration was 25 μM, and TDP-43 concentration was ~0.23 μM. The averaged data (n=8) and standard deviation are plotted. Source data are provided as a Source Data file.

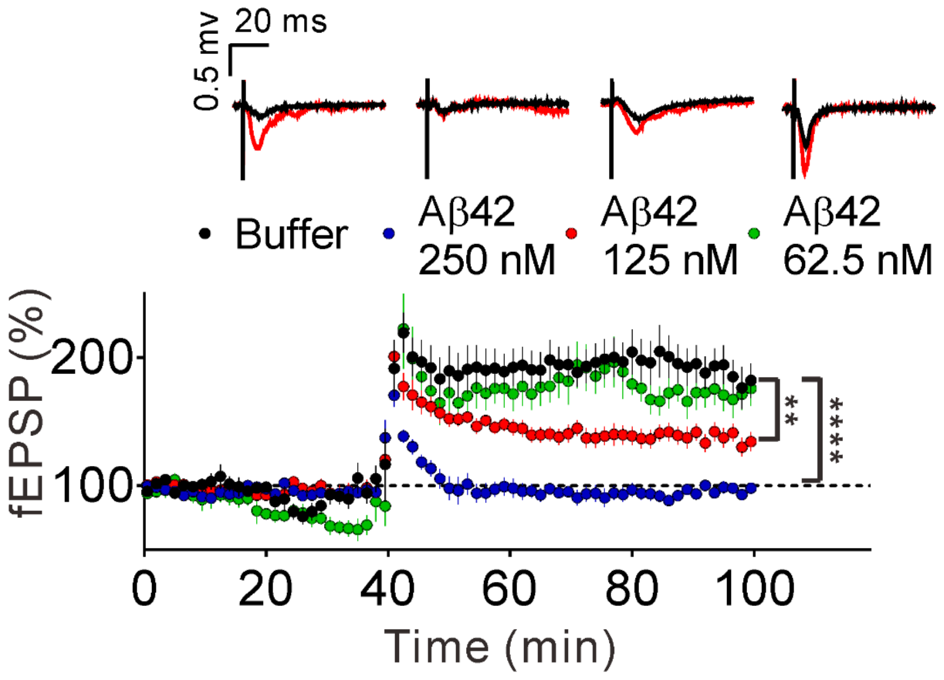

Supplementary Figure 5. Aβ42 impairs the hippocampus LTP dose-dependently. Field EPSPs (fEPSPs) were measured from a Schaffer collateral fiber on a hippocampal slice from C57BL/6 mice. After recording was stabilized for 10 min, the slices were treated with buffer control (black) or different concentrations of Aβ42 at 250 nM (blue), 125 nM (red), and 62.5 nM (green) for 30 min. After the treatment, the hippocampal slices were subjected to a theta burst stimulation (black arrowhead) to induce LTP. Scale bar, 0.5 mv, 20 ms. For Aβ42 concentration analysis, the mice brain slices were treated with buffer control (n=9, independent slices), 250 nM Aβ42 (n=10, independent slices), 125 nM Aβ42 (n=9, independent slices), and 62.5 nM Aβ42 (n=5, independent slices). The averaged data and s.e.m. are plotted. Each group of fEPSPs before (black line) and after (red line) the theta burst stimulation is shown individually in the upper panel. Aβ42 versus buffer, repeated two-way ANOVA, $**p < 0.01$, $****p < 0.0001$ (Buffer vs. Aβ42-125 nM, $p = 0.004$; Buffer vs. Aβ42-250 nM, $p < 0.0001$). Source data are provided as a Source Data file.

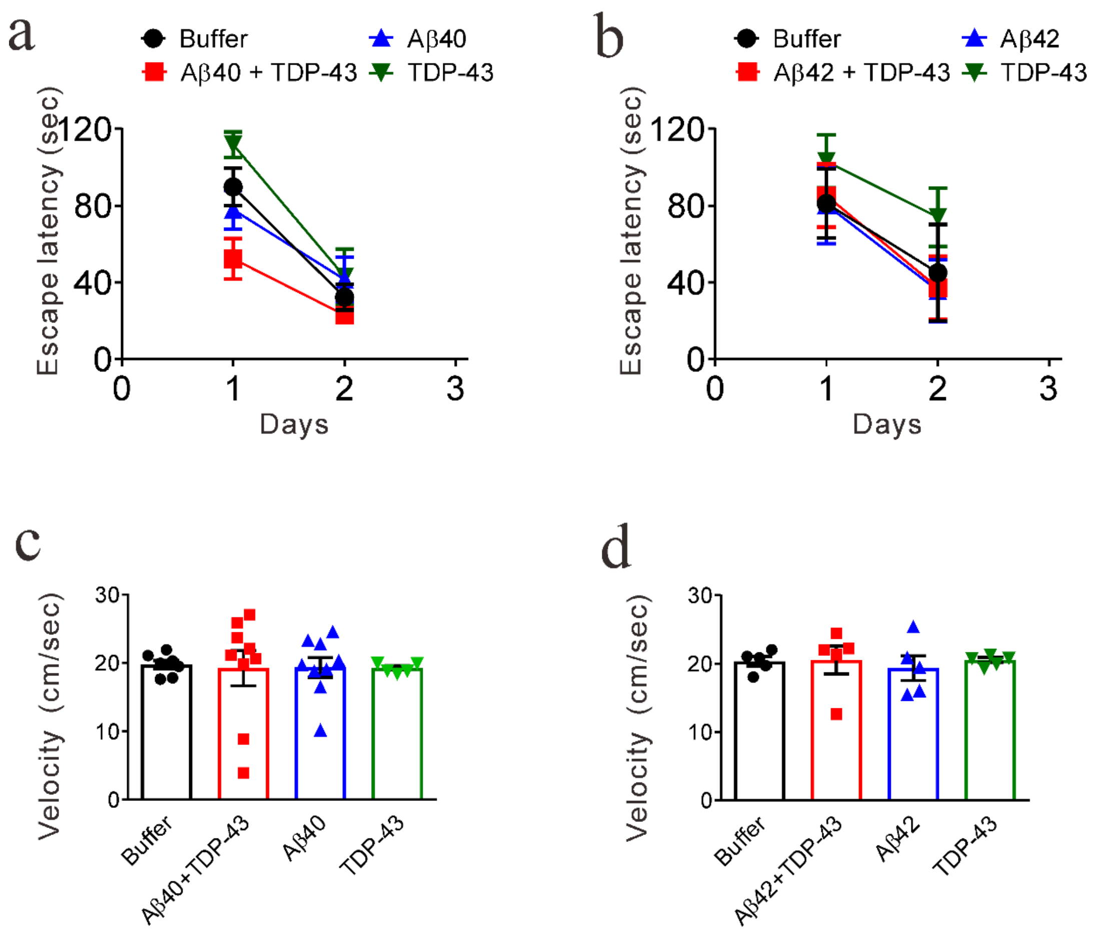

Supplementary Figure 6. The TDP-43-induced Aβ species does not impair the visible ability and navigate motivation in WT mice. The spatial learning function of the protein-injected mice was determined by MWM. Pre-training phase for (a) Aβ40 and (b) Aβ42. Escape latency is the time to find the visible platform in the pre-training phase. (c-d) The swimming speed for Aβ40 and Aβ42 injected mice, respectively. The visible ability and navigate motivation were not impaired in each mouse. The sample size is as described in Fig. 5. The data were colored for buffer control (black), Aβ and TDP-43 (red), Aβ (blue), and TDP-43 (green). The averaged data and s.e.m. are plotted. Source data are provided as a Source Data file.

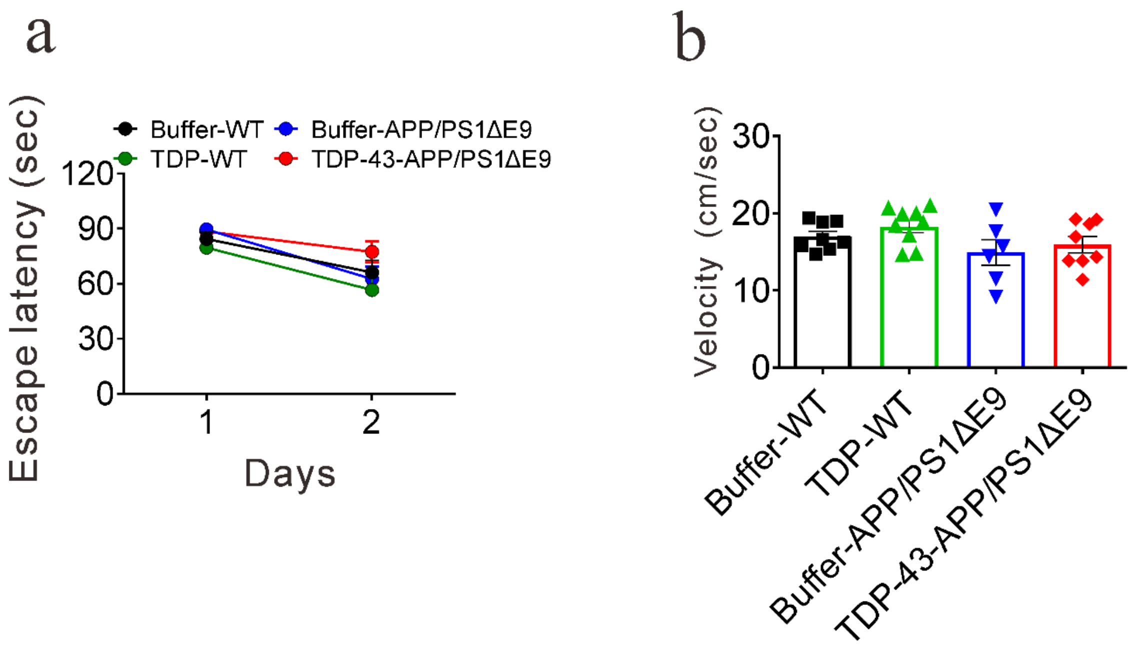

Supplementary Figure 7. TDP-43 or buffer injection does not impair the visible ability and navigate motivation in WT and APP/PS1ΔE9 mice. The spatial learning function of the mice was determined by MWM. (a) Pre-training phase for the WT and APP/PS1ΔE9 mice received buffer or TDP-43 injection. Escape latency is the time to find the visible platform in the pre-training phase. (b) The swim speed velocity. The sample size is as described in Fig. 6. The data were colored for buffer-injected WT mice (black), TDP-43-injected WT mice (green), buffer-injected APP/PS1ΔE9 mice (blue), and TDP-43-injected APP/PS1ΔE9 mice (red). The averaged data and s.e.m. are plotted. Source data are provided as a Source Data file.

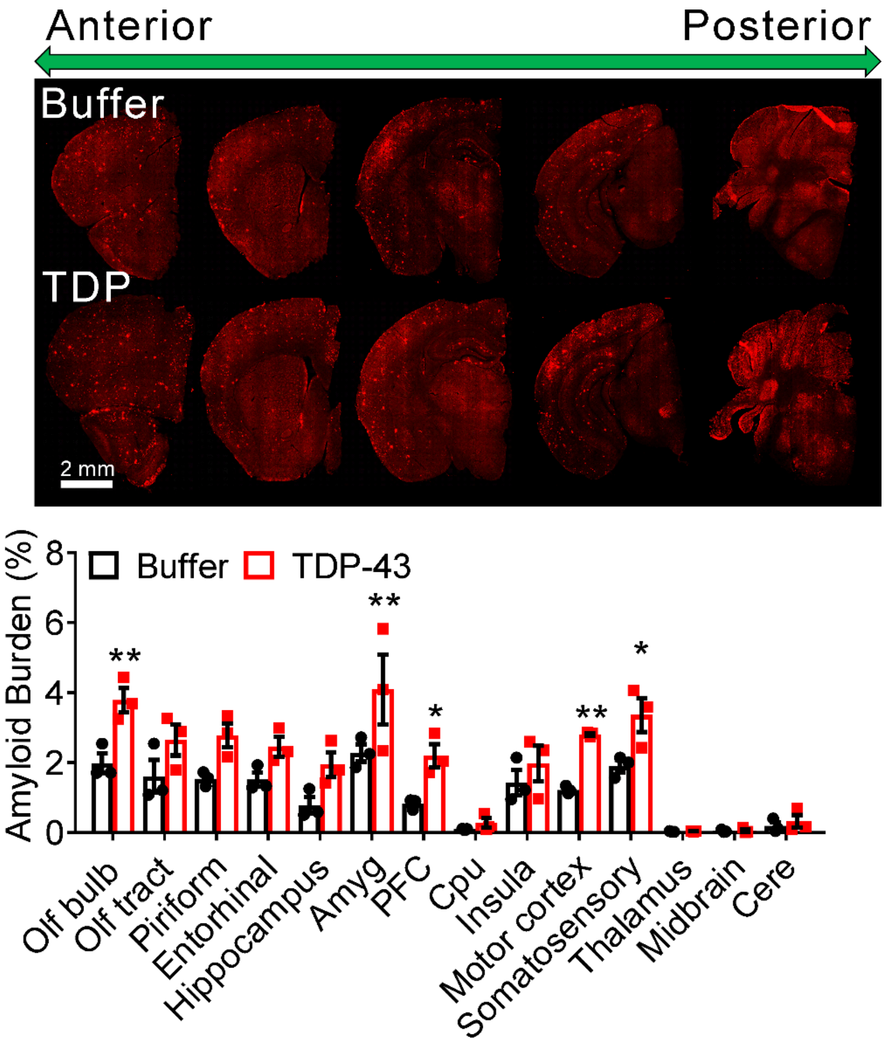

Supplementary Figure 8. TDP-43 injection increases amyloid plaque burden in APP/PS1ΔE9 mice. Buffer (n=3 independent mice) or TDP-43 (n= 3 independent mice) was injected to APP/PS1ΔE9 mice at 5 months of age and the brains were collected one month after injection for immunostaining. Representative immunofluorescence staining of amyloid plaques from the anterior to posterior coronal brain sections and the quantitative results in different brain regions were shown (PFC, prefrontal cortex; Cpu, caudate-putamen; Cere, cerebellum). The averaged data and s.e.m. are plotted. The statistical analysis was performed by Repeated two-way ANOVA, Bonferroni's post-hoc test, TDP-43 vs respective Buffer, *$p < 0.05$, **$p < 0.01$, ***$p < 0.001$ (Olfactory bulb, Buffer vs. TDP-43, $p = 0.0017$; Amygdala: Buffer vs. TDP-43, $p = 0.0017$; Prefrontal cortex: Buffer vs. TDP-43, $p = 0.0279$; Motor cortex: Buffer vs. TDP-43, $p = 0.0069$; Somatosensory cortex: Buffer vs. TDP-43, $p = 0.0158$). Source data are provided as a Source Data file.

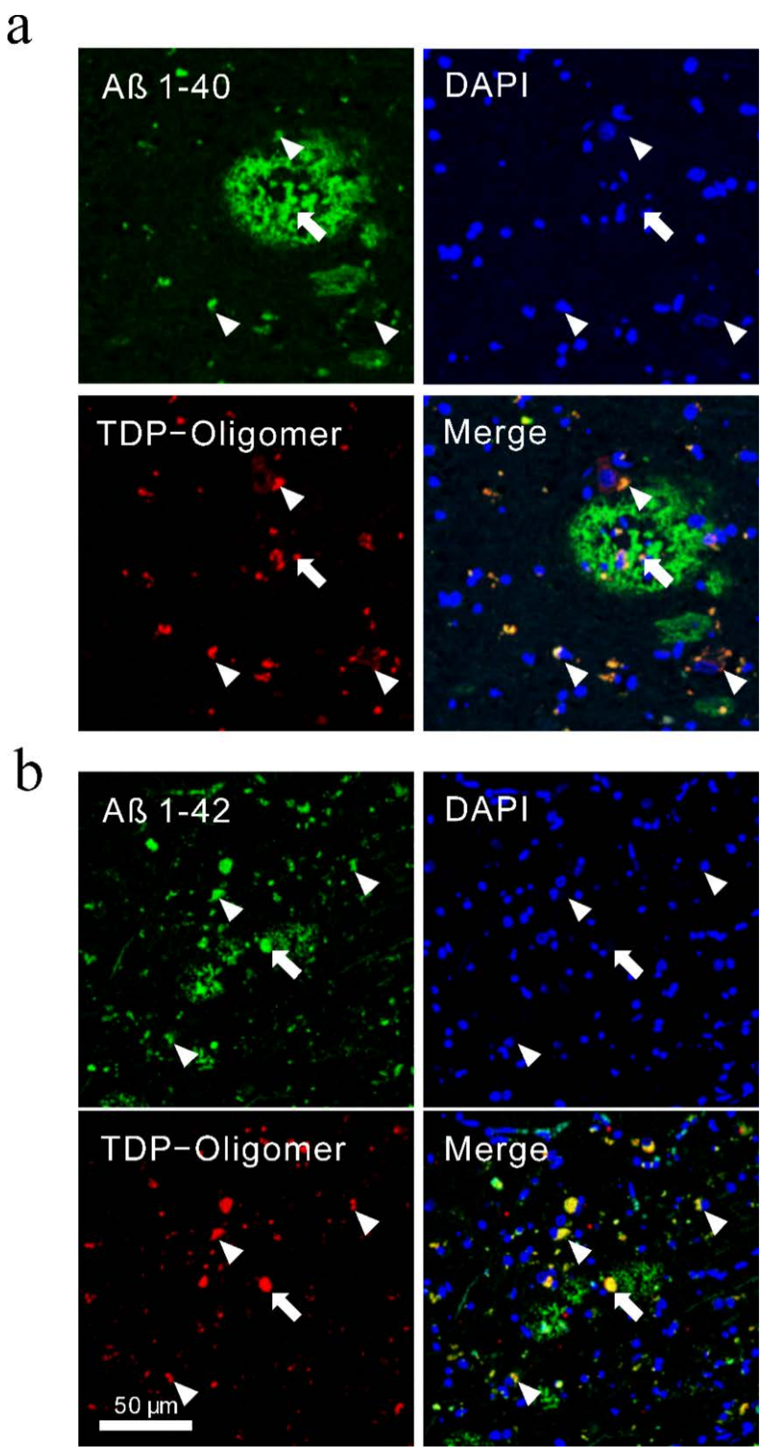

Supplementary Figure 9. TDP-43 oligomers colocalize with Aβ40 and Aβ42 in the brain of an AD patient. Representative immunostaining micrographs reveal that TDP-43 oligomers colocalized with intraneuronal Aβ (arrowhead) and partly with amyloid plaques (arrow) in the entorhinal cortex of a 77-year old Braak stage IV AD patient. (a) Aβ40. Aβ40 specific antibody 11A50 was used. (b) Aβ42. Aβ42 specific antibody 12F4 was used. Four induvial samples were examined. Source data are provided as a Source Data file.

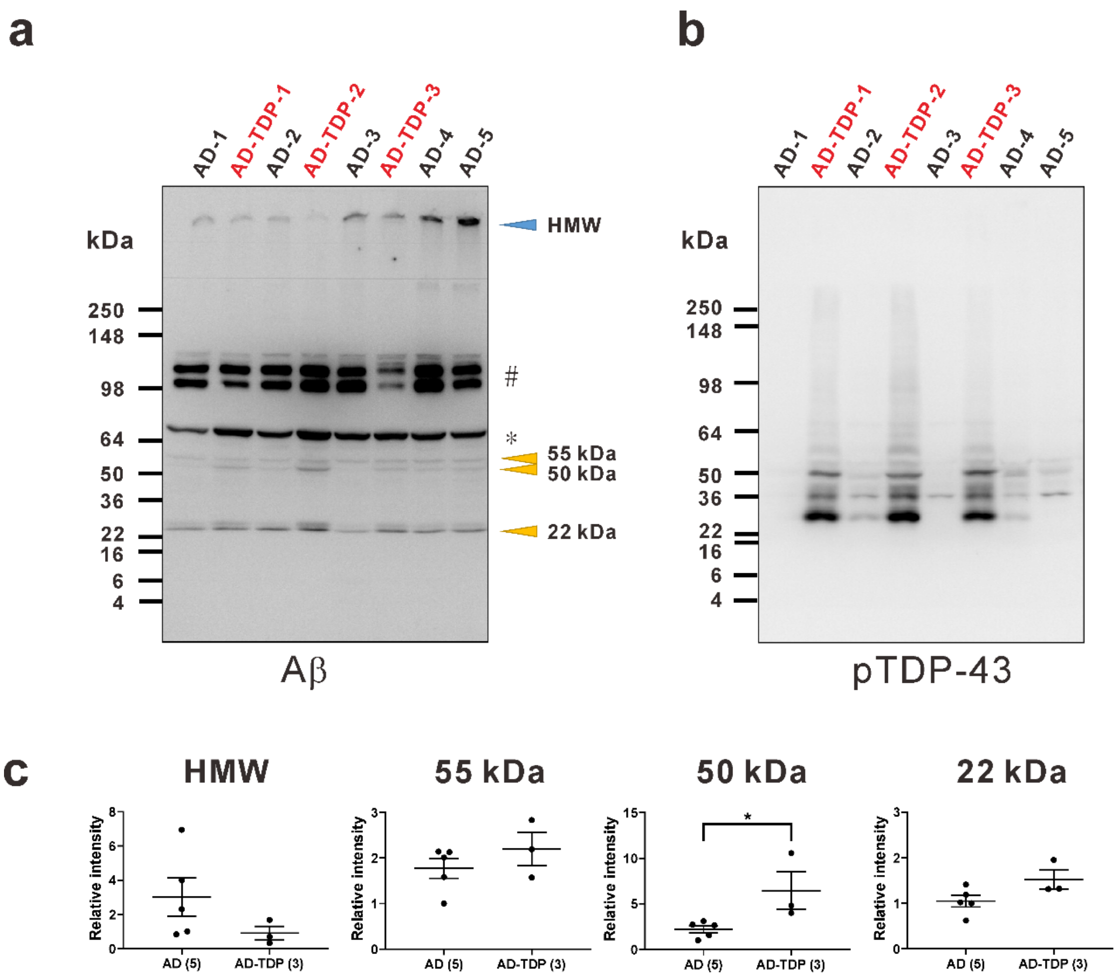

Supplementary Figure 10. Aβ oligomers increased in AD hippocampi carrying TDP-43 pathology. Representative Western blot images of (a) extracellular-enriched fraction and (b) urea fractions of AD hippocampi. (c) Quantification of different Aβ assembly in the extracellular-enriched fractions. The Aβ assembly in the hippocampus tissues of AD patients with (AD-TDP, n=3) or without (AD, n=5) hyperphosphorylated TDP-43 was examined by western blot with anti-Aβ antibody 6E10/4G8. TDP-43 pathology indicated by hyperphosphorylated TDP-43 was detected by phosphorylated TDP-43 antibody. High-molecular-weight (HMW) aggregates and Aβ oligomers, 55, 50, 22 kDa, were indicated by arrow heads (*, non-specific bands; # secreted APP). The averaged data and s.e.m. are plotted. The presence of 50 kDa Aβ oligomers were significantly higher in AD patients carrying TDP-43. The statistical analysis was performed by two-tailed Mann-Whitney test, *$p < 0.05$ ($p = 0.0357$). Source data are provided as a Source Data file.

Supplementary References

1 Uryu, K. *et al.* Concomitant TAR-DNA-binding protein 43 pathology is present in Alzheimer disease and corticobasal degeneration but not in other tauopathies. *J Neuropathol Exp Neurol* **67**, 555-564 (2008).

2 Fang, Y. S. *et al.* Full-length TDP-43 forms toxic amyloid oligomers that are present in frontotemporal lobar dementia-TDP patients. *Nat Commun* **5**, 4824 (2014).
