# Supplementary material for: TDP-43 interacts with amyloid-β, inhibits fibrillization, and worsens pathology in a model of Alzheimer’s disease
Source: Nat Commun. 2020 Nov 23;11:5950. doi: 10.1038/s41467-020-19786-7 (PMC7683652; doi:10.1038/s41467-020-19786-7)
Supplement: Supplementary file 3 — Reporting Summary [file 41467_2020_19786_MOESM3_ESM.pdf]

nature research

Corresponding author(s): Yun-Ru Chen

Last updated by author(s): Oct 15, 2020

Reporting Summary

Nature Research wishes to improve the reproducibility of the work that we publish. This form provides structure for consistency and transparency in reporting. For further information on Nature Research policies, see Authors & Referees and the Editorial Policy Checklist .

Statistics

For all statistical analyses, confirm that the following items are present in the figure legend, table legend, main text, or Methods section.

n/a	Confirmed	
☐	☒	The exact sample size (*n*) for each experimental group/condition, given as a discrete number and unit of measurement
☐	☒	A statement on whether measurements were taken from distinct samples or whether the same sample was measured repeatedly
☐	☒	The statistical test(s) used AND whether they are one- or two-sided<br>*Only common tests should be described solely by name; describe more complex techniques in the Methods section.*
☐	☒	A description of all covariates tested
☐	☒	A description of any assumptions or corrections, such as tests of normality and adjustment for multiple comparisons
☐	☒	A full description of the statistical parameters including central tendency (e.g. means) or other basic estimates (e.g. regression coefficient) AND variation (e.g. standard deviation) or associated estimates of uncertainty (e.g. confidence intervals)
☐	☒	For null hypothesis testing, the test statistic (e.g. *F*, *t*, *r*) with confidence intervals, effect sizes, degrees of freedom and *P* value noted<br>*Give P values as exact values whenever suitable.*
☒	☐	For Bayesian analysis, information on the choice of priors and Markov chain Monte Carlo settings
☒	☐	For hierarchical and complex designs, identification of the appropriate level for tests and full reporting of outcomes
☒	☐	Estimates of effect sizes (e.g. Cohen's *d*, Pearson's *r*), indicating how they were calculated

Our web collection on statistics for biologists contains articles on many of the points above.

Software and code

Policy information about availability of computer code

Data collection	For ThT: The data were collected by SoftMax Pro 6.3 in SpectraMax M5, Molecule Devices<br>For CD: The spectra were collected by Spectra Manager 2.0 in Jasco J-815 spectropolarimeter<br>For Western blot: ImageQuart was used<br>For ELISA: The reading was collected by SoftMax Pro 6.3 in SpectraMax M5, Molecule Devices<br>For Octet: The signals were obtained by Octet RED96 system with the Data Analysis Software (ForteBio).<br>For animal behavior Study: The behavior studies were recorded and analyzed by EthoVision video tracking system (Noldus Information Technology, Wageningen, Netherlands)<br>For LTP: The fEPSP were recorded by MED–P515A/5 (1 mm) probe (Alpha MED Scientific Inc., Osaka, Japan) with Alpha MED64 MEA system.<br>For IHC: The images were taken by Aperio AT2 Digital Pathology Scanner (Leica Biosystem, Mannheim, Germany)<br>For IF: The images were taken by Aperio FL Digital Pathology Scanner (Leica Biosystem, Mannheim, Germany).<br>For Abeta assembly and IP: The blots were detected by Imagequant LAS4000 system (GE Healthcare, Life sciences, Hungary).<br>For human Staining: The images were taken by Aperio CS2 Digital Pathology Scanner (Leica Biosystem).
Data analysis	For Octet Red96, KD value was obtained by data fitting using the Data Analysis Software (ForteBio).<br>For animal Study: The behavior data were recorded and analyzed by EthoVision video tracking system (Noldus Information Technology, Wageningen, Netherlands)<br>For LTP: The fEPSP slopes were quantified by MED64 Mobius Software<br>For IHC: The images were analyzed by ImageJ 1.8.0_60 (NIH, USA)<br>For IF: The images were analyzed by ImageJ 1.8.0_60 (NIH, USA)<br>For Abeta assembly and IP: The blotting signal was quantified by ImageJ 1.8.0_60 (NIH, USA).<br><br>All data except for Octet data were further plotted by GraphPad Prism version 7.0.

GraphPad Prism version 7.0. was used for statistical analysis.

For manuscripts utilizing custom algorithms or software that are central to the research but not yet described in published literature, software must be made available to editors/reviewers. We strongly encourage code deposition in a community repository (e.g. GitHub). See the Nature Research guidelines for submitting code & software for further information.

Data

Policy information about availability of data

All manuscripts must include a data availability statement. This statement should provide the following information, where applicable:

- Accession codes, unique identifiers, or web links for publicly available datasets
- A list of figures that have associated raw data
- A description of any restrictions on data availability

Data supporting the findings of this manuscript are available from the corresponding authors upon reasonable request. A reporting summary for this Article is available as a Supplementary Information file. Source data are provided with this paper.

Field-specific reporting

Please select the one below that is the best fit for your research. If you are not sure, read the appropriate sections before making your selection.

[x] Life sciences [ ] Behavioural & social sciences [ ] Ecological, evolutionary & environmental sciences

For a reference copy of the document with all sections, see nature.com/documents/nr-reporting-summary-flat.pdf

Life sciences study design

All studies must disclose on these points even when the disclosure is negative.

Sample size

The sample sizes in each study were described in the manuscript. To determine the size, we referenced the previous study to determine the sample size1 according to the previous study and suggested by the Academia Sinica Institutional Animal Care and Utilization Committee (IACUC). The minimum require sample of each group is 4 for protein injection study to against the null hypothesis. Previous study indicated that APP/PS1ΔE9 (B6C3-Tg (APPswe, PSEN1dE9) 85Dbo/Mmjax mice have 20% mortality around 5-month-old2, so we used more than double sample size from 4 to 10 for the transgenic mice injection study.

References

1. Arifin, W. N. & Zahiruddin, W. M. Sample Size Calculation in Animal Studies Using Resource Equation Approach. The Malaysian journal of medical sciences : MJMS 24, 101-105 (2017).

2 Gimbel, D. A. et al. Memory impairment in transgenic Alzheimer mice requires cellular prion protein. The Journal of neuroscience : the official journal of the Society for Neuroscience 30, 6367-6374 (2010).

Data exclusions

No data was excluded

Replication

All the data with error bars were collected from the independent mice (from 3 to 10, each group/study). We used the individual mice number as independent experiment repeat times.

Randomization

Allocations are randomized in the experiments

Blinding

All animal studies were blinded including the electrophysiology study for group allocation and data collection during the experiments. The data were then analyzed without blinding since we need to know their condition to analyze them in specific groups.

Reporting for specific materials, systems and methods

We require information from authors about some types of materials, experimental systems and methods used in many studies. Here, indicate whether each material, system or method listed is relevant to your study. If you are not sure if a list item applies to your research, read the appropriate section before selecting a response.

Materials & experimental systems

n/a	Involved in the study	
[ ]	[x]	Antibodies
[x]	[ ]	Eukaryotic cell lines
[x]	[ ]	Palaeontology
[ ]	[x]	Animals and other organisms
[ ]	[x]	Human research participants
[x]	[ ]	Clinical data

Methods

n/a	Involved in the study	
[x]	[ ]	ChIP-seq
[x]	[ ]	Flow cytometry
[x]	[ ]	MRI-based neuroimaging

Antibodies

Antibodies used

All antibodies used were described in 'Methods' section of the manuscript and supplementary information. The detailed information is listed in validation.

Validation

Anti-β-Amyloid, 1-16 (6E10) Mouse mAb, BioLegend, Cat No 803002, Lot No. B226151. Application: WB, Direct ELISA, IHC-P, IHC-F, EM, and ICC. Reactivity: Hu. 173 citations. BioLegend website antibody validation: 1) Western blot analysis of human Aβ1-40 peptide, Aβ1-42 peptide, recombinant human APP751 protein, and human brain lysate shows the 6E10-positive bands. 2) IHC staining on the formalin-fixed paraffin-embedded normal human and Alzheimer's disease brain tissues shows the presence of 6E10-positive Aβ plaques in Alzheimer's disease brains, which are absent in normal controls. Relevant literature reports: 1) IF analysis of the Aβ plaques in the brain tissues of a human APP-transgenic mouse model shows the labeling of the plaque core by 6E10 (Aging Cell 2016, p953–963).

Anti-β-Amyloid, 17-24 Antibody (4G8) Mouse mAb, BioLegend, Cat No 800702, Lot No. B249633. Application: IHC-P, Direct ELISA, IHC-F, ICC, and IP. Reactivity: Hu. 57 Citations. BioLegend website antibody validation: 1) IHC staining of the formalin-fixed paraffin-embedded human Alzheimer's disease brain tissue shows the presence of 4G8-positive Aβ plaques. Relevant literature reports: 1) IF analysis of the Aβ plaques in the brain tissues of a human APP-transgenic mouse model shows the labeling of the plaque core by 4G8 (Aging Cell 2016, 15, p953–963). 2) Western blot analysis of the brain tissue extracts from an AD mouse model shows 4G8-positive Aβ-oligomer bands (Journal of Alzheimer's Disease 2019, 70, p487–503).

Anti-Aβ40 specific antibody (11A50-B10) Mouse mAb, BioLegend, Cat No. SIG-39140. Lot No. B236987. 10 Citations. Application: IHC. Reactivity: Ms. BioLegend website validation: 1) The IHC staining of formalin-fixed paraffin-embedded Alzheimer's disease brain tissue shows that the presence of Aβ plaques detected by anti-β-Amyloid, 1-40 antibody (11A50-B10). Relevant literature reports: 1) IF analysis with 11A50-B10 shows that vascular Aβ accumulation was present in the retinal pericytes in Alzheimer's disease patients, but was absent in cognitively normal controls (Acta Neuropathologica 2020, 139, 813–836).

Anti-Aβ42 specific antibody (12F4) Mouse mAb, BioLegend, Cat No. SIG-39142. Lot No. B267487. 17 Citations. Application: IHC-P, Direct ELISA, WB, IHC-F, ELISA. Reactivity: Hu, Ms, Rt. BioLegend website validation: 1) IHC analysis of 12F4 shows that the presence of Aβ plaques in Alzheimer's disease brains, which were absent in normal controls. Relevant literature reports: 1) IF analysis with 12F4 shows that vascular Aβ42 deposits were detected inside retinal pericytes in Alzheimer's disease patients but not in cognitively normal controls (Acta Neuropathologica 2020, 139, 813–836).

Anti-Oligomer (A11) Rabbit pAb, ThermoFisher Scientific, Cat No. AHB0052, Lot No. TB260305. 64 Citations. Application: Dot blot, ELISA, IHC, WB, ICC, IF, IP, Neutralization. Reactivity: Hu, Ms, Rt. ThermoFisher Scientific website validation: 1) Aβ42 oligomers, IAPP oligomers, and α-Synuclein oligomers can be detected by a dot blot analysis using A11, but not Aβ42 monomers, and Aβ42 fibrils.

Anti-Amyloid Fibrils (OC) Rabbit pAb, Merck, Cat No. AB2286, Lot No. 2424776. 37 Citations. Application: IP, ICC, IHC, ELISA, WB, DB. Reactivity: Hu. Merck website validation: 1) OC shows stronger reactivity with amyloid fibrils, shows weaker reactivity with monomers and displayed no reactivity with prefibrillar oligos. in the dot blot assay.

Anti-TDP-43 (C-Terminal) Rabbit pAb, Proteintech, Cat No. 12892-1-AP, Lot No. 00050567. Application: WB, IP, IHC, IF, chIP, and ELISA. Reactivity: Hu, Ms, Rt, Mk, Dr et al. 106 Citations. Proteintech website validation: 1) TDP-43 protein in Hela cell lysates can be immunoprecipitated by the TDP-43 antibody 12892-1-AP. 2) WB analysis of TDP-43 protein in sh-Control and sh-TDP-43 transfected A594 cells shows the intensity of the TDP-43 band (~40 kDa) was reduced in sh-TDP-43 transfected cells.

Anti-TDP-43 Rabbit pAb, Proteintech, Cat No. 10782-2-AP, Lot No. 00052103. Application: WB, RIP, IP, IHC, IF, IEM, FC, CoIP, chIP, and ELISA. Reactivity: Hu, Ms, Rt, Zf et al. 1098 Citations. Proteintech website validation: 1) TDP-43 protein in HeLa cell lysates can be immunoprecipitated by the TDP-43 antibody 10782-2-AP. 2) WB analysis of TDP-43 protein in sh-Control and sh-TDP-43 transfected HeLa cells shows the intensity of the TDP-43 band (~40 kDa) was reduced in sh-TDP-43 transfected cells.

Anti-TDP-43, phospho Ser409/410 Mouse mAb (11-9), Cosmo Bio USA, Cat No. CAC-TIP-PTD-M01, Lot No. 11-9-20. 38 Citations. Application: ELISA, IHC(f), WB. Reactivity: Hu. Proteintech website validation: 1) WB analysis of FTLD-U patient tissues shows the phosphorylated full-length TDP-43 at 45 kDa, -25 kDa fragments and smearing substances, while the phosphorylated TDP-43 bands were disappeared in the WB result of protein phosphatase pretreated FTLD-U tissue lysates detected by phosphorylated TDP-43 antibody (11-9). 2) IHC analysis shows nucleocytoplasmic inclusions presented in dentate gyrus of FTLD-U were specifically stained by the phosphorylated TDP-43 antibody (Clone11-9).

Anti-Iba1 Rabbit pAb, GeneTex, Cat No. GTX100042, Lot No. 39476. Application: WB, ICC/IF, IHC-P, IHC-Fr, FACS, IHC. Reactivity: Hu, Ms, Rat. 25 Citations. GeneTex website antibody validation: 1) Iba1 antibody detects Iba1 protein on the paraffin-embedded rat hindbrain by IHC analysis.

Anti-MAP-2 Guinea pig pAb, Synaptic Systems, Cat No.188 004, Lot No. 3-3C. 82 Citations. Application: WB, IP, ICC, IHC, IHC-P/FFPE. Reactivity: Hu, Rt, Ms. Synaptic Systems website validation:1) The neuron-specific cytoskeletal protein MAP-2 in the cultured hippocampus neurons can be detected by IF analysis with anti-MAP2. 2) The neurons can be detected by indirect immunostaining of a PFA fixed paraffin-embedded mouse brain section with anti-MAP2.

Anti-GAPDH Mouse mAb, GeneTex, Cat No. 627408. Lot No. 41323. 202 Citations. Application: WB, ICC/IF, IHC-P, EMSA. Reactivity: Hu, Ms, Rt, Zf et al. GeneTex website validation: 1) Anti-GAPDH Ab can detect GAPDH protein in 293T cells, NIH-3T3 cell lysates mouse brain lysates, PC-12 cells, and rat brain lysates.

Animals and other organisms

Policy information about studies involving animals; ARRIVE guidelines recommended for reporting animal research

Laboratory animals	C57BL/6 mice were obtained from the National Laboratory Animal Center (Taipei, Taiwan). The APP/PS1DeltaE (B6C3-Tg (APPswe,PSEN1dE9)85Dbo/Mmjax) mice were obtained from the Jackson Laboratory, USA. Sex and age were described in the manuscript and supplementary information.
Wild animals	No wild animals were used in the study
Field-collected samples	No field collected samples were used in the study.
Ethics oversight	All experiments were done in accordance with National Institutes of Health Guideline for animal research (Guide for the Care and Use of Laboratory Animals) and Taiwan Animal Protection Law and approved by the Academia Sinica Institutional Animal Care and Utilization Committee (IUCAC 16-02-939)

Note that full information on the approval of the study protocol must also be provided in the manuscript.

Human research participants

Policy information about studies involving human research participants

Population characteristics	1) Human AD brain tissue sections for immunostaining. Age at death; Gender; Diagnosis: Patient 1: 77 years; male; Braak stage IV. Patient 2: 71 years; male; Braak stage IV. Patient 3: 84 years; male; Braak stage V. Patient 4: 70 years; female; Braak stage V. 2) Source of human hippocampus extracts for western blot. Age at death; Gender; Diagnosis: AD-TDP-1: 89 years; female; Alzheimer's disease (Thal Phase 5, Braak Stage V, CERAD 3), cerebrovascular disease atherosclerosis in Circle of Willis, small vessel disease, diffuse pallor in periventricular white matter, microinfarct in middle frontal gyrus, and hemorrhage (2mm) in cerebellum vessel occlusion in white matter. /AD-TDP-2: 92 years; female; Alzheimer's Disease (Thal Phase 5, Braak Stage VI, CERAD 3) with TDP-43 cytoplasmic inclusions and mild cerebral amyloid angiopathy. /AD-TDP-3: 82 years; female; Path report not finalized. /AD1: 88 years; male; Path report not finalized. /AD2: 91 years; male; Alzheimer's disease (Thal Phase 5, Braak stage VI, CERAD 3) and ischemic brain injury. /AD3: 88 years; female; Alzheimer's disease (Thal Phase 4, Braak stage V, CERAD 3), mild cerebral amyloid angiopathy, and moderate atherosclerosis. /AD4: 86 years; female; Alzheimer's disease (Thal Phase 5, Braak stage VI, CERAD 3), amygdala predominate Lewy body pathology, cerebral amyloid angiopathy, and white matter pathology (pallor and arteriolosclerosis). /AD5: 78 years; female; Alzheimer's disease (Thal Phase 5, Braak stage VI, CERAD 3), severe cerebral amyloid angiopathy, ischemic brain injury, and amygdala predominate Lewy body pathology.
Recruitment	The samples were post mortem brain collection from Alzheimer's Disease Center, University of California, Davis. Participants were recruited and consented in the Alzheimer's Disease Center at University of California, Davis. All of the human tissue-related procedures and usage were approved by the Human Subjects Research Ethics, Academia Sinica, Taiwan.
Ethics oversight	Alzheimer's Disease Center, University of California, Davis, Sacramento, California

Note that full information on the approval of the study protocol must also be provided in the manuscript.
